# Activation of NF-κB signaling via cytosolic mitochondrial RNA sensing in kerotocytes with mitochondrial DNA common deletion

**DOI:** 10.1038/s41598-021-86522-6

**Published:** 2021-04-01

**Authors:** Xin Zhou, Ludvig J. Backman, Patrik Danielson

**Affiliations:** 1 Department of Integrative Medical Biology, Umeå University, 90187 Umeå, Sweden; 2 Department of Clinical Sciences, Ophthalmology, Umeå University, Umeå, Sweden; 3 Department of Community Medicine and Rehabilitation, Physiotherapy, Umeå University, 90187 Umeå, Sweden

**Keywords:** Cell biology, Immunology, Molecular biology

## Abstract

Scar formation as a result of corneal wound healing is a leading cause of blindness. It is a challenge to understand why scar formation is more likely to occur in the central part of the cornea as compared to the peripheral part. The purpose of this study was to unravel the underlying mechanisms. We applied RNA-seq to uncover the differences of expression profile in keratocytes in the central/peripheral part of the cornea. The relative quantity of mitochondrial RNA was measured by multiplex qPCR. The characterization of mitochondrial RNA in the cytoplasm was confirmed by immunofluoresence microscope and biochemical approach. Gene expression was analyzed by western blot and RT qPCR. We demonstrate that the occurrence of mitochondrial DNA common deletion is greater in keratocytes from the central cornea as compared to those of the peripheral part. The keratocytes with CD have elevated oxidative stress levels, which leads to the leakage of mitochondrial double-stranded RNA into the cytoplasm. The cytoplasmic mitochondrial double-stranded RNA is sensed by MDA5, which induces NF-κB activation. The NF-κB activation thereafter induces fibrosis-like extracellular matrix expressions and IL-8 mRNA transcription. These results provide a novel explanation of the different clinical outcome in different regions of the cornea during wound healing.

## Introduction

Scar formation during corneal wound healing is a significant clinical problem. It is the fourth leading cause of blindness globally according to the World Health Organization (https://www.who.int/blindness/publications/globaldata/en/), accounting for about ~ 4% of all cases of avoidable vision loss. The only available curative treatment for corneal blindness at present, is transplantation surgery. However, since corneal transplants are not without complications and since there is sometimes a lack of donor grafts, it is important to increase the knowledge about the pathophysiological mechanisms of corneal wound healing to find ways to avoid scar formation. Among ophthalmologists specializing in the cornea, it is known that post-surgical scar formation in the periphery of the cornea—for instance after penetrating keratoplastic transplantation—is sparse, whereas the central part of the cornea is more prone to develop scars following trauma or infection. Furthermore, as the central part of the cornea is in the field of vision, scar formation in this area may result in a major visual problem for the patient.

It is known that the expression profiles of the collagen-synthesizing stromal cells (keratocytes) of the cornea are significantly different between keratocytes derived from the peripheral cornea as compared to those from the central cornea^1^. The expression of keratocyte markers (keratocan, lumican, CD34, and ALDH) are relatively higher in the central cornea while the cells in the peripheral cornea appear to be more characterized as stem cells^2,3^. The corneal stromal stem cells are important for corneal wound healing and have been successfully used to prevent fibrotic scar formation^2,4^. The extracellular matrix (ECM) production is crucial for wound healing and its disruption leads to scarring and opacities^5^. However, since no differences in ECM-related genes from keratocytes of different regions have been reported, this implies that other factors are of relevance.

The central part of the cornea is the region of the eye that is most exposed to UV radiation^6^. A UVA-induced transcriptomic and proteomic study of corneal stroma showed that cumulative UVA exposure causes changes in extracellular matrix^7^. Mitochondrial DNA (mtDNA) common deletion (CD) is a marker of chronic UV radiation in the skin^8^. The deletion is caused by the loss of a large fragment of mtDNA from nucleotide positions 8482-13459 bp. mtDNA CD is also found to be selectively concentrated in the central cornea and gradually decreases towards the periphery^9^. These findings suggest that ECM differences seen between central and peripheral cornea could be caused by UV radiation, which in turn affects mtDNA CD content.

mtDNA CD engulfs more than 1/3 of mtDNA, which can cause specific defects in complex I, IV and V of the electron transport chain (ETC). CD-induced ETC defects results in (1) increased oxidative stress and (2) mitochondrial reactive oxygen species (ROS) generation^10–12^. Oxidative stress can result in damage of the mtDNA, which subsequently results in an inflammatory response^13^. The immunostimulatory potential of mtDNA was first reported by Collins et al. in 2004^14^. Since then, several studies have shown that mtDNA is also associated with induction of damage-associated molecular patterns (DAMPs) that even further enhance pro-inflammatory responses^15–18^. DAMPs are endogenous molecules that are released from damaged cells or organelles, including mitochondria. DAMPs, including protein, metabolites, DNA and RNA, can activate the innate immune system and are considered to have a pathogenic role in inflammatory diseases. Inflammation is a fundamental process in corneal wound healing, but excessive corneal inflammation can result in an increased profibrotic response with more corneal opacification^19–21^. DAMPs can cause persistent inflammation in cells^22^. The chronic inflammatory response may exacerbate tissue damage and eventually result in scar formation^23^.

Mitochondrial ROS generation can promote mtDNA release and induce DAMPs^24,25^. However, whether mitochondrial ROS also can cause mtRNA leakage is unknown. Dhir et al. first reported that mtRNA can activate the immune response driven by melanoma differentiation-associated protein 5 (MDA5) antiviral signaling pathway^26^. MDA5 belongs to the retinoic acid-inducible gene-I-like receptors receptor (RLR) family, which serves as a pattern recognition receptor to recognize double-strand RNA (dsRNA). Stimulation of MDA5 leads to the activation of NF-κB and type I inferferon response. It is also known that mtRNA helicase SUV3 and polynucleotide phosphorylase PNPase prevent the formation and release of mitochondrial double-stranded RNA into the cytoplasm. Interestingly, PNPase is also involved in reducing oxidative RNA damage and protecting against oxidative stress^27,28^. It is intriguing to study whether oxidative stress can promote mtRNA release to induce innate immune responses.

Inflammatory response plays an important role in corneal wound healing. Prolonged inflammation may not only compromise the wound healing, but also worsen the scar formation^29,30^. Proinflammatory cytokines released from keratocytes can recruit immune cells after corneal injury. Interleukin 8 (IL-8) is a key mediator in the migration of neutrophils to the sites of injury^31^. Uncontrolled release of various proteases by neutrophils may result in stromal degradation and ulceration, which might result in corneal opacity and neovascularization^32^.

We hypothesize that the central cornea suffers more from UV radiation, as indicated by the occurrence of mtDNA CD. The keratocytes with CD could suffer from elevated oxidative stress that leads to excessive mtDNA/RNA release into the cytoplasm. The released mtDNA/RNA can induce endogenous DAMPs and subsequent inflammation, which would ultimately exacerbate the scar formation pathogenesis in the cornea.

In the present study, we confirm that there are different levels of mtDNA CD in the central and peripheral cornea. We further confirm that IL-8 expression is exclusively regulated by NF-κB signaling, which is activated by released mtRNA via MDA5 signaling. Our results provide a potential explanation for the different outcomes of scar formation in the central and peripheral cornea. Moreover, the obtained results could also have a profound medical implication of corneal scar formation.

## Results

### Keratocytes with mitochondrial DNA common deletion have elevated oxidative stress and IL-8 expression

**Figure 1:**
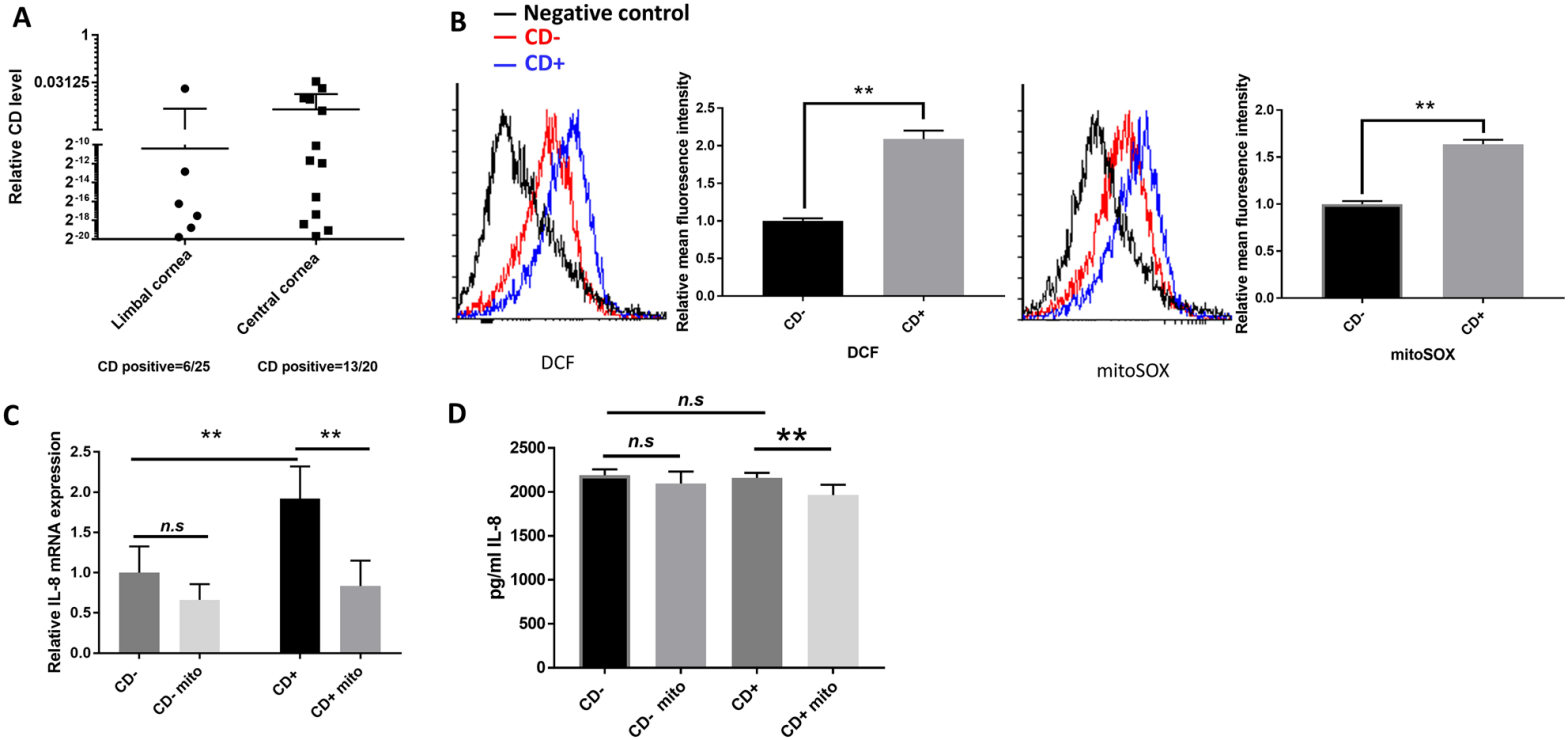
Oxidative stress in CD+ keratocytes up-regulates the IL-8 expression. (**A**) CD/total mtDNA ratios in 45 human subjects quantified by multiplex-qPCR. 26 values were zero and are not included on the graph. (**B**) Flow cytometric detection of DCFH-DA and mitoSOX signal in CD+/− keratocytes (*n* = *3*). (**C**) mRNA expression of IL-8 in CD+/− keratocytes. Cells either treated with 10 μM mitoTEMPO (Mito), or not, for 24 h (*n* = 3). (**D**) Quantification of secreted IL-8 level using ELISA. Cells treated, or not treated, with 10 μM mitoTEMPO for 24 h (*n* = 3). Values are means ± SD. n.s. (not significant); *P < 0.05, **P < 0.01.

There was a higher occurrence of CD formation in the keratocytes from central cornea as compared to the limbal (i.e. peripheral) cornea (Fig. 1A). Here cells with CD were termed CD+ keratocytes, and cells without detectable CD were termed CD− keratocytes. Out of 25 samples of limbal cornea, 6 samples were CD+ , while 13 out of 20 samples of central cornea were CD+ . CD+ keratocytes showed an increased level of intracellular oxidative stress as compared to CD− keratocytes, as indicated by a ~ 2.0 fold difference in mean fluorescence intensity observed in DCF signal (Fig. 1B). Superoxide production (mitoSOX) in mitochondria in CD+ keratocytes was ~ 1.6 fold higher in mean fluorescence intensity than in CD− keratocytes (Fig. 1B). IL-8 is known to enhance keratocyte migration and recruit neutrophils, which are two necessary steps in the corneal wound healing^33^. Oxidative stress has been reported to upregulate IL-8 gene expression^34^. As expected, IL-8 expression, as measured at mRNA level, was higher in CD+ keratocytes as compared to in CD− keratocytes. There was no significant difference of IL-8 at extracellular secretion level measured by ELISA. Administration of mitoTEMPO significantly reduced the level of IL-8 expression in CD+ keratocytes (Fig. 1C-D), as well as the oxidative level in mitochondria (Additional Data Fig. 1), suggesting a positive correlation between mitochondrial ROS and IL-8 expression.

### MDA5 signaling pathway induces NF-kappaB activation in CD+ keratocytes

**Figure 2:**
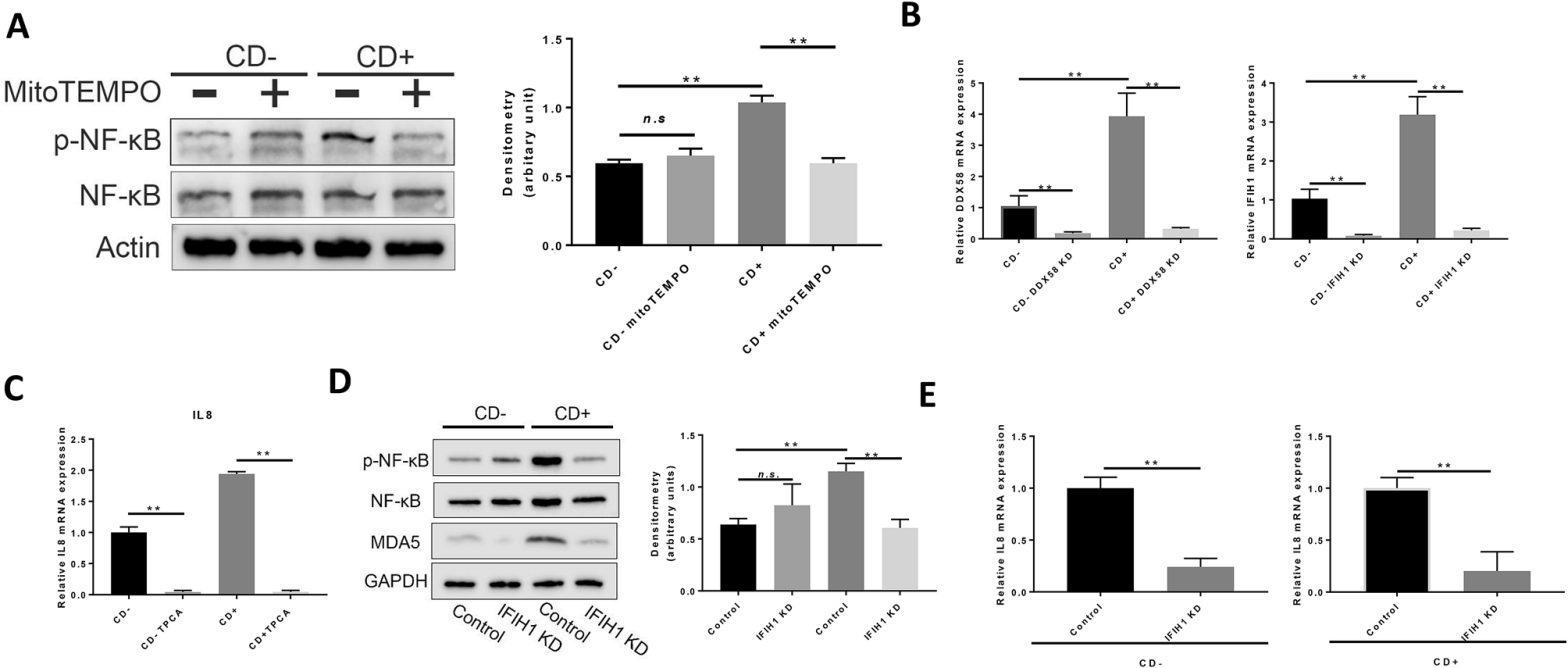
MDA5 signaling induces IL-8 upregulation via NF-κB phosphorylation in CD+ keratocytes. (**A**) Western blot of NF-κB phosphorylation in CD− and CD+ keratocytes with/without mitoTEMPO (*n* = 4). NF-κB and p-NF-κB were blotted and cropped from the same gel. p-NF-κB was blotted first and the membrane was stripped and washed for NF-κB blotting. Actin was blotted separately from a different gel using the corresponding samples. Exposures were adjusted automatically by the Odyssey Fc imaging system. (**B**) DDX58 and IFIH1 mRNA expression in CD+/− keratocytes 48 h after respective siRNA transfection (*n* = 3). (**C**) IL-8 mRNA expression in keratocytes 24 h after 20 nM TPCA treatment (*n* = 3). (**D**) NF-κB phosphorylation, analyzed by western blot, after IFIH1 knock-down in CD+/− keratocytes (*n* = 3). NF-κB and p-NF-κB were blotted and cropped from the same gel. p-NF-κB was blotted first and the membrane was stripped and washed for NF-κB blotting. GAPDH and MDA5 were blotted separately from a different gel using the corresponding samples. Exposures were adjusted automatically by the Odyssey Fc imaging system. (**E**) IL-8 mRNA expression in CD+/− keratocytes after IFIH1 knockdown by siRNA transfection (*n* = 3). Values are means ± SD. n.s. (not significant); *P < 0.05, **P < 0.01.

The RNA-sequencing analysis was performed to uncover the signaling pathway that might connect oxidative stress to IL-8 upregulation. There were 4485 genes that were differently expressed in CD+ keratocytes compared to in CD− keratocytes (Additional Data Fig. 2A). Kyoto Encyclopedia of Genes and Genomes (KEGG) pathway enrichment analysis revealed several alternated pathways that were upregulated in CD+ keratocytes as compared to in CD− keratocytes. Interestingly, several enriched pathways were related to virus infection and immune response (Additional Data Fig. 2B). As we found increased IL-8 expression in CD+ keratocytes as compared to in CD− keratocytes, the KEGG enrichment pathway indicated that IL-8 upregulation could be regulated by the cytosolic RNA sensing pathway (Additional Data Fig. 2C). As shown in the diagram, RNA sensing mediated by RIG-I/MDA5 could transduce the signal to activate NF-κB, which in turn induce IL-8 up-regulation. To confirm this, we measured the phosphorylated NF-κB in CD+ keratocytes, combined with the treatment of mitoTEMO. Corresponding to the pattern of IL-8 expression, NF-κB phosphorylation was higher in CD+ keratocytes, and this was abolished by mitoTEMPO treatment (Fig. 2A), as was the case for IL-8. DDX58 and NFKBIA mRNA expression were consistent with the RNA-seq data (Fig. 2B, Additional Data Fig. 2D), while IFIH1 mRNA expression was higher in CD+ keratocytes as compared to in CD− keratocytes, which was not detected by RNA-seq data (Fig. 2B). TPCA1, an inhibitor of NF-κB nuclear localization, completely abrogated IL-8 mRNA expression (Fig. 2C), confirming a direct role of NF-κB in IL-8 regulation^35–38^.

MDA5 expression was significantly reduced after siRNA transfection, as confirmed by qPCR (Fig. 2B) and western blot (Fig. 2D) in both CD+/− keratocytes. DDX58 mRNA expression was reduced 48 h after siRNA transfection (Fig. 2B) but the corresponding RIG-I expression was barely detectable by western blot using two antibodies (MA5-31715, Invitrogen; 3743S, Cell signaling) (data not shown). siRNA-mediated MDA5 depletion (Fig. 2D) resulted in a marked reduction of p-NF-κB in CD+ keratocytes, which was accompanied by a down-regulation of IL-8 mRNA expression (Fig. 2E). These results show that IL-8 expression in CD+ keratocytes was mediated by NF-κB via MDA5 signaling.

### Mitochondria double-stranded RNA (mtdsRNA) escapes from mitochondria in CD+ keratocytes

**Figure 3:**
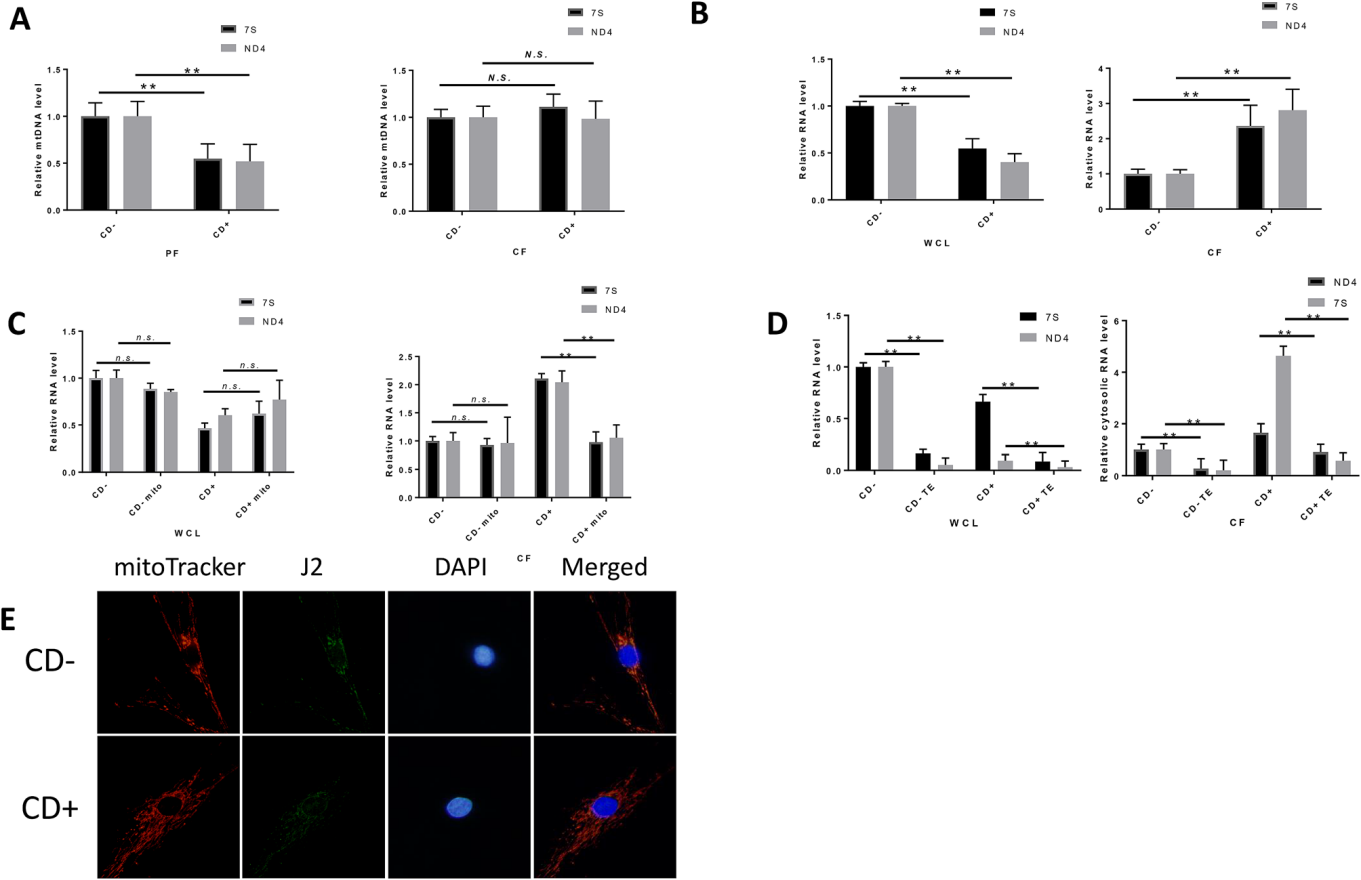
Higher level of mtdsRNA in the cytoplasm of CD+ keratocytes compared to in that of CD− keratocytes. (**A**) The relative level of mtDNA in the pellet fraction (PF) or cytosolic fraction (CF) measured by multiplex qPCR (*n* = 3). (**B**) Relative mtRNA in whole-cell lysate (WCL) and CF of CD+/− keratocytes measured by multiplex qPCR (*n* = 3). (**C**) Relative mtRNA in CF of CD+ keratocytes 24 h after 10 μM mitoTEMPO treatment (*n* = 3). (**D**) Mitochondrial RNA level with/without Terminator 5′-phosphate-dependent exonuclease treatment (TE) in WCL and CF (*n* = 3). (**E**) Representative fluorescence image of dsRNA in CD+/− cells. mitoTracker (red), J2 foci (greed) and DAPI (blue) were merged in the right column (merged). Values are means ± SD. n.s. (not significant); *P < 0.05, **P < 0.01.

The stimulation of NF-κB can be induced by cytosolic DNA/RNA. Many studies have shown that cytosolic DNA induces NF-κB activation^39–41^. We first tried to determine whether cytosolic DNA was the source to induce NF-κB activation. Cytosolic nuclear DNA signal, as measured by qPCR using reference gene β2M and GAPDH probe, was close to background level (the negative control), indicating no leakage of nuclear DNA in the cytoplasm (data not shown). The source of DNA in the cytoplasm therefore could be from mitochondria. A multiplex PCR approach was applied to measure the cytosolic mtDNA content. Reduced mtDNA copy number in mitochondria was found in CD+ cells compared to in CD− keratocytes (Fig. 3A), whereas the cytosolic mtDNA content was at the same level in CD+/− keratocytes. These results contradict the possibility that cytosolic DNA triggered the immune response in CD+ keratocytes. Recent studies found that also mtRNA can induce innate immune response^26,42^. To determine whether mtRNA triggered an immune response in CD+ keratocytes, total mtRNA and cytosolic mtRNA were quantified using multiplex qPCR. The total mtRNA in CD− keratocytes was higher than that in CD+ keratocytes (Fig. 3B). This could be due to the lower mtDNA copy number in CD+ keratocytes compared to CD− keratocytes. Contrary, a significantly higher amount of mtRNA in the cytoplasm was found in CD+ keratocytes compared to in CD− keratocytes. Administration of mitoTEMPO markedly reduced the cytosolic cotent of mtRNA in CD+ keratocytes, while it has no significant effects on cytosolic mtRNA in CD− keratocytes, nor in the whole cell fraction (Fig. 3C). Dhir et al. found that mitochondria double-stranded RNA (mtdsRNA), which escaped from mitochondria into the cytosol, was a source to trigger antiviral signaling via MDA5 signaling^26^. Alternatively, RNA polymerase III (Pol III) can use cytosolic mtDNA as template to produce single-stranded mtRNA (mtssRNA) that can induce RIG-I-mediated signaling^42^. Terminator 5′-Phosphate-Dependent Exonuclease (TE) is an exonuclease that can digest RNA that have a 5′ monophosphate but cannot digest RNA having a 5′-triphosphate. RNA transcribed in mitochondria lacks 5′-cap protection from TE, while Pol III-transcribed RNA has a 5′-triphosphate cap. TE treatment can thus distinguish RNA transcribed in mitochondrial from RNA transcribed by Pol III in the cytoplasm. The level of mtRNA was significantly reduced after TE digestion both in the whole-cell and in the cytosolic fraction (Fig. 3D). RNA isolated from mitochondrial fraction can be completely digested by TE treatment, resulting in no detectable signal in qPCR (data not shown). Consistently, immunostaining of dsRNA with anti-dsRNA (J2) showed that J2 foci were mostly co-localized with mitochondria in both CD+/− keratocytes (Fig. 3E). Treatment of TE before J2 staining resulted in markedly reduction of J2 foci signal in keratocytes (Additional Data Fig. 3C). These results suggest that the majority of cytosolic mtRNA is from the mitochondrial matrix in CD+ keratocytes.

### Altered extracellular matrix (ECM) expressions in CD+ myofibroblasts are linked to mitochondrial ROS and NF-kappaB signaling

**Figure 4:**
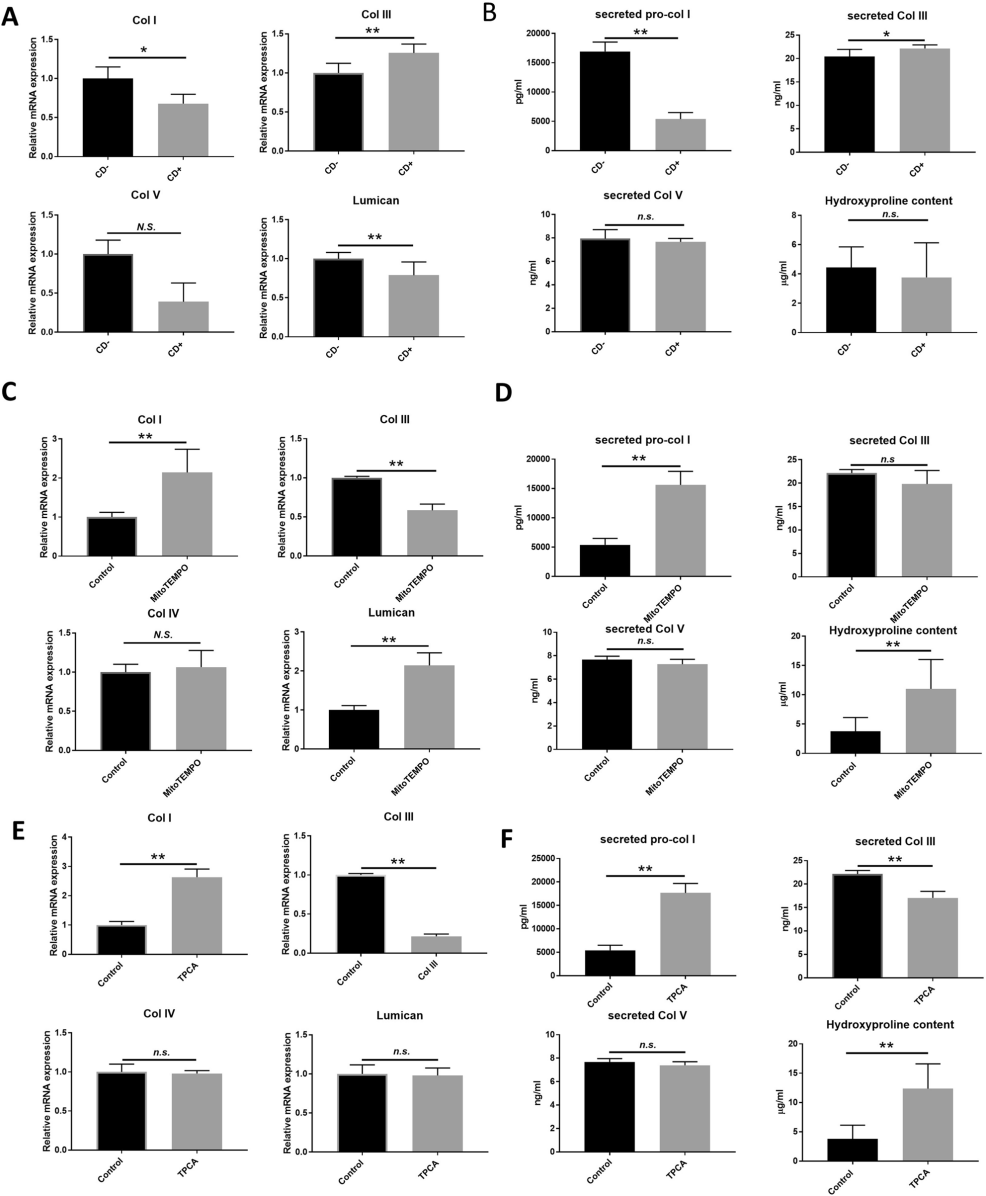
Changes in various ECM components in corneal myofibroblasts are dependent on mitochondrial ROS and NF-κB signaling. (**A**) RT qPCR on the mRNA expression of COL1A1, COL3A1, COL5A1 and lumican in corneal myofibroblasts (*n* = 3). (**B**) Secretion of pro-collagen I, collagen III, collagen V, and hydroxyproline content in supernatant collected 2 d after fibrosis induction (*n* = 3). (**C**) RT qPCR of the mRNA expression of COL1A1, COL3A1, COL5A1 and lumican in CD+ corneal myofibroblasts. Cells were treated with 10 µM mitoTEMPO for 2 d (*n* = 3). (**D**) Secretion of pro-collagen I, collagen III, collagen V, and hydroxyproline content in supernatant collected 2 d after fibrosis induction in CD+ myofibroblasts simultaneously treated with 10 µM mitoTEMPO (*n* = 3). (**E**) RT qPCR of the mRNA expression of COL1A1, COL3A1, COL5A1 and lumican in CD+ corneal myofibroblasts treated with 20 nM TPCA for 48 h (*n* = 3). (**F**) Secretion of pro-collagen I, collagen III, collagen V, and hydroxyproline content in supernatant collected 2 days after fibrosis induction in CD+ myofibroblasts simultaneously treated with 20 nM TPCA (*n* = 3). Values are means ± SD. n.s. (not significant); *P < 0.05, **P < 0.01.

RNA-seq data showed that col3a1 expression was significantly up-regulated in CD+ cells (Additional Data Fig. 2A). This result prompted us to study whether there were differences in collagen expression in CD+/− keratocytes, since collagen expression and organization are vitally important for corneal wound healing and also key factors altered in scar formation. Keratocytes were transformed into myofibroblasts in an in vitro fibrosis model^43^, as identified by the expression of α-SMA (Additional Data Fig. 4). mRNA expression of COL1A1, COL5A1 and lumican were lower in CD+ myofibroblasts compared to CD− myofibroblast (Fig. 4A). Pro-col I secretion from CD+ myofibroblast was significantly reduced as compared to from CD− myofibroblast (Fig. 4b). Col III secretion and mRNA expression were higher in CD+ myofibroblasts as compared to CD− myofibroblasts (Fig. 4A, top right panel, and Fig. 4B, top right panel, respectively). ECM deposition, as measured by hydroxyproline assay, showed no significant difference between CD− and CD+ myofibroblasts (Fig. 4B). To study whether mitochondrial oxidative stress and NF-κB signaling are involved in the collagen expression patterns in CD+ myofibroblasts, mitoTEMPO or TPCA treatment was used. Both mitoTEMPO and TPCA treatment effectively reversed the mRNA expression and secretion of Col I and Col III, as well as hydroproline content (Fig. 4C-F). These results show that mitochondrial ROS and NF-κB signaling participate in the regulation of collagen expression and ECM depostion in CD+ myofibroblasts.

## Discussion

CD (common deletion) has been reported to be concentrated in the central cornea as compared to in the limbal (peripheral) region^9^. Our results confirmed that a higher occurrence of CD is seen in central corneal tissue as compared to in limbal cornea. Keratocytes in the central cornea are more exposed to UV radiation compared to limbal keratocytes^6^, which means a higher risk of CD induction. As discussed by Gendron et al.^9^, the eyelid can cover most of the cornea in response to different light conditions but the central part is always the most exposed region of the eye^6^. Despite the observation of CD in the corneal tissue, the functional consequences of mtDNA deletion in keratocytes have not been reported. Our results showed an increase of mitochondrial superoxide, as well as a general elevation of oxidative stress, in CD+ keratocytes. However, it is not known whether the oxidative stress originated from mitochondria could affect the corneal wound healing process.

Previously we have found that IL-8 enhances keratocyte migration and neutrophil recruitment^33^, which are necessary steps during corneal wound healing. However, if the neutrophil infiltration in an injured cornea is not tightly controlled, the release of various proteases may lead to stromal degradation and ulceration, which in turn might result in corneal opacity and neovascularization^32^. It is interesting to note that ROS has been positively correlated to IL-8 expression^44–46^. Our results confirmed that mitochondria generated ROS upregulates IL-8 expression, at both mRNA level and in secretion, by specific mitochondrial superoxide scavenger. Approximately 90% of the cellular ROS are generated in the mitochondria through OXPHOS^47^. mtDNA CD ablates several mitochondrial genes encoding for ETC. The elevated mitoSOX signal in CD+ keratocytes is thus expected (Fig. 1B), since the deficiency of ETC leads to the increased generation of ROS^48,49^. However, the mechanism behind the overall increased oxidative stress in CD+ keratocytes needs further investigation.

IL-8 is encoded by the CXCL8 gene. Many factors are involved in the transcription regulation of CXCL8, such as NF-κB^38^, CAAT/enhancer-binding protein β (C/EBPβ)^50^, cAMP response element-binding protein (CREB)^51^, and activating protein 1 (AP-1)^52^. Among all the transcription factors in CXCL8 regulation, NF-κB is closely interacting with ROS (reviewed in^53^). ROS can directly oxidize the cysteine in the Rel-homology domain (RHD) of NF-κB to inhibit its DNA binding^54–56^. In contrast, ROS can also activate NF-κB through alternative IκBα phosphorylation^57,58^.

Besides the regulation of NF-κB by targeting related proteins directly, ROS also induces NF-κB activation indirectly by inducing DNA/RNA release into the cytoplasm. Mitochondrial ROS accumulation has been found to promote mtDNA release and initiate inflammation^24,25^. It is possible that mtRNA also escape from mitochondria in the same manner as mtDNA. Reports have shown that cytosolic RNA, from different sources, triggers the immune response. Suspene et al. proposed that cytoplasmic mtDNA can be transcribed by pol III and trigger RIG-I mediated IFN expression^42^. They found transfected mtDNA upregulated type I interferon expression in a human myeloid cell line. Dhir et al. found that mitochondrial dsRNA that had escaped into the cytoplasm in a PNPase-dependent manner triggers antiviral signaling^26^. Our RNA-seq data indicate the potential involvement of RIG-I-like receptor signaling pathway in NF-κB signaling in CD+ keratocytes (Additional Data Fig. 2). RIG-I-like receptor signaling can either be initiated by RIG-I or MDA5 RNA sensing. RIG-I specifically binds to 5′-pppRNA and short dsRNA, while MDA5 recognizes long dsRNA. Both receptors might be responsible for the induction of IL-8 in CD+ keratocytes. Both IFIH1 and DDX58 expression were higher in CD+ keratocytes as compared to that in CD− keratocytes (Fig. 2B). However, RIG-I protein was not detectable in any of the two types of keratocytes, whereas MDA5 protein level was more abundant in CD+ keratocytes (Fig. 2D), corresponding to its elevated level of IFIH1 mRNA. Notably, knockdown of IFIH1 led to reduced IL-8 mRNA in both CD+/− keratocytes (Fig. 2E), suggesting a regulatory role of MDA5 in IL-8 expression. Taken together, we confirmed that IL-8 upregulation requires the accumulation of ROS in the mitochondria and expression of MDA5, suggesting that dsRNA sensing via MDA5 accounts for the elevated IL-8 expression in CD+ keratocytes.

We found the presence of mtRNA in the cytoplasm in keratocytes. Similarly, background cytosolic dsRNA was also found in HeLa cells using J2 immunofluorescence staining^26^. Although we found quantitative differences of cytosolic mtRNA by qPCR in CD+/− keratocytes, the differences were not detectable by J2 staining. The method based on immunofluorescence staining might not be sensitive enough to detect subtle changes in cytosolic dsRNA content. The higher expression of mtRNA in the qPCR data, might also be explained by the mitochondria in CD+ keratocytes leaking more, since the RNA-seq data also showed that several genes involved in mPTP were upregulated (VDAC1, VDAC2, SLC25A4 and SLC25A5). Overall, the concomitant reduction of cytosolic mtdsRNA (Fig. 3E), IL-8 expression (Fig. 1C,D), and NF-κB phosphorylation (Fig. 2A) suggest that cytosolic mtdsRNA could activate NF-κB signaling in a mitochondrial ROS-dependent manner. It is interesting to note that IL-8 has been reported to induce NF-κB activation in a dose-dependent manner in different cell types^59^. Presumably, a positive feedback loop of IL-8–NF-κB might prolong the inflammatory state in CD+ cells in an autocrine manner.

Mitochondrial ROS generation has been found to promote mtDNA release^24,25^. Szczesny et al. found that low-level oxidative stress can also induce mtDNA oxidation and an related inflammatory response^13^. Notably, increased expression of cytoplasmic RNA sensors, such as DDX58 and IFIH1, were found in Tfam heterozygous knockout mouse cells^60^, which increase the innate immune response. These findings indicate that mtRNA escapes from mitochondria as mtDNA does. The liberation of mtRNA from mitochondria was recently reported^26^. Interestingly, they found that the release of mtdsRNA is dependent on Polynucleotide phosphorylase (PNPase). PNPase is an exoribonuclease primarily located in mitochondria^61^. It has been shown to remove oxidatively damaged RNA with high affinity^27,28,62^. There might be more mtdsRNA escape in cells under mitochondrial oxidative stress, if the capacity of PNPase is not increased correspondingly.

mtDNA mutation such as CD has been proposed as a molecular marker for photoaging of skin^63,64^ and cornea^9^. Photoaging is often referred to as a premature phenotype in skin caused by repeated exposure to ultraviolet (UV) radiation. Lower expression of type I pro-collagen has been found in the fibroblasts of photoaged skin compared to naturally aged skin^65^. Similarly, markedly reduced expression of collagen I (COL1A1) and collagen V (COL5A1 and A2) was observed in keratocytes treated by chronic UVA exposure as a model of photoaging^7^. Lumican, a protein that is responsible for the regulation of collagen assembly, is also downregulated in the in vitro photoaging model of corneal stroma keratocytes^7^. Collagen III is expressed weakly in the cornea under normal physiological conditions^66^. Its expression increases greatly during wound healing or inflammation and is markedly upregulated in corneal scars^67^. The elevated collagen III expression might be due to inflammation, which should be abolished—at least in later stages of wound healing—to prevent corneal scar formation. Taken together, the expression profile of CD+ cells resembles a photoaging phenotype. Considering the significant reduction of Col I expression and upregulation of Col III in photoaged keratocytes, corneal photoaging should be considered as a potential risk factor to the outcome of corneal wound healing and scar formation.

The different expression profiles of collagens in CD+/− cells suggest the potential role of NF-κB in corneal scar formation. NF-κB signaling has been reported to regulate collagen expression patterns. NF-κB activation inhibits expression of the COL1A1^68^. Conversely, reduced NF-κB activity resulted in a significant increase in COL1A1 gene expression^69^. On the opposite, Col III expression is increased with NF-κB signaling is activated^70,71^. Our results revealed the negative regulation of collagen secretion by NF-κB and mitochondrial ROS (Fig. 4 C-F). However, there was no significant difference of the actual ECM deposition between CD− and CD+ myofibroblasts (Fig. 4 B, lower right panel), indicating there were other factors determining the total collagen synthesis in CD+ myofbibroblast. Overall, targeting mitochondrial redox status and/or preventing pathological NF-κB activation during corneal wound healing could be a potential approach to counter-act corneal scar formation.

## Conclusion

It is well-known that the central part of the cornea is more likely to form a scar than peripheral parts. This is especially of concern since a central corneal scar can result in a major visual problem for the patient. To be able to prevent or reduce the incidence of scar formation in the central cornea is therefore of great clinical importance. Our findings provide a novel pathway to explain the onset of permanent corneal scarring/fibrosis post-surgery or trauma. Moreover, the obtained results could also have a profound medical impact beyond corneal tissue, as a new link between mtDNA deletions and scar formation is confirmed.

## Materials and methods

### Isolation and culture of human keratocytes

Isolation and culture of primary keratocytes were performed as previously described^1^. Healthy human corneal tissue was received for research purpose from the Tissue Establishment, Eye Bank Umeå, at the University Hospital of Umeå, Sweden. The tissue originated from deceased individuals who had chosen, when alive, to donate their corneas postmortem for transplantation and research, according to Swedish law, and grafts not used for transplantation, or the leftover tissue from healthy grafts used for transplant surgery, were delivered to the laboratory for research purpose. An overview of the donor information (mean age and age range; sex) is shown in Table 1. The Regional Ethical Review Board in Umeå reviewed the study and determined it to be exempt from the requirement for approval (2010‐373‐31 M). The study followed the principles of the Declaration of Helsinki.

**Table 1:** Donor information.

Cornea origin	N	Mean age	Age range	Male	Female
Central	20	71.9	26–89	n = 14	n = 6
Limbal	25	70.0	26–89	n = 20	n = 5

Cell isolation and culture were performed as described previously^1,33,72^. Corneal samples were scraped using a sterile scalpel to remove any remaining epithelial or endothelial cells, before being washed in sterile Hanks' balanced salt solution (Invitrogen, Carlsbad, CA). The remaining stromal layer was cut into 1–2 mm^2^ pieces with a scalpel and then digested with 2 mg mL^−1^ collagenase (Sigma, St. Louis, MO) overnight at 37 °C. The suspension was centrifuged and the pellet was cultured in DMEM/F‐12 media (Gibco, Carlsbad, CA) supplemented with 2% fetal bovine serum (FBS; Gibco, Carlsbad, CA) and 1% penicillin–streptomycin (Invitrogen, Carlsbad, CA) and placed in a humidified incubator at 37 °C with 5% CO_2_. Media was changed every third day until the cells reached confluence.

### In vitro human corneal fibrosis model

The in vitro human corneal fibrosis model was adapted from Karamichos et al.^73^ and has been successfully established in our lab^43^. Corneal fibroblasts were plated on plastic culture dishes at desired densities in DMEM/F-12 10% FBS medium and stimulated by a stable vitamin C derivative L-Ascorbic acid 2-phosphate sesquimagnesium salt hydrate (VitC; Sigma-Aldrich, St. Louis, MO, USA, # A8960) at a concentration of 0.5 mM, and 0.25 ng/ml recombinant human TGF-β1 (R&D Systems, # 240-B).

### Separation of cytosolic DNA

Confluent cells in 10 cm dish were harvested by centrifugation (2 min, 500 g). Cells were washed twice with ice cold PBS, before resuspended in 1 ml homogenization buffer (40 mM Tris–HCl, 25 mM NaCl, 5 mM MgCl_2_, 20 ug/ml digitonin) and incubated 5 min on ice. After the homogenization, 125 μL of equilibration buffer (400 mM Tris–HCl, 250 mM NaCl, 50 mM MgCl_2_) was added. The homogenate was centrifuged at 1200 g at 4 °C for 3 min to pellet the nuclear fraction (NF). The supernatant was collected and centrifuged at 20,000 g at 4 °C for 10 min to pellet the mitochondrial fraction (MF) and supernatant were used as cytosolic fraction (CF). NF and CF were combined and subjected to DNA extraction and subsequent multiplex qPCR for cytosolic mtDNA quantification.

### DNA isolation

Total DNA was isolated by PureLink Genomic DNA kit (Invitrogen, Hilden, Germany) according to the manufacturer's protocol. For cytosolic DNA isolation, 20 μL Proteinase K and 20 μL RNase A were added to 200 μL of supernatant and incubated at 55 °C for 30 min and then heated at 95 °C for 5 min. The mixture was then cooled down at room temperature and mixed with same volume of 100% ethanol. The mixture was transferred to a PureLink Spin Column and centrifuged for 15 s at > 8000 g. The flow-though was discarded. These steps were repeated to increase the DNA concentration. Washing and elution were then performed according to the standard protocols.

### Separation of cytosolic RNA

The cell homogenate was prepared using the protocol for cytosolic DNA separation. The homogenate was centrifuged at 20,000 g at 4 °C for 10 min and the cytosolic fraction (CF) were used for RNA extraction (c.f. RNA isolation) and subsequent multiplex qPCR. To test the purity of cytosolic RNA in the CF extraction, the proteins from whole cell lysate (WCL), CF and pellet fraction (PF) were blotted using GAPDH, Histone H3 and TFAM antibody. No nuclear and mitochondrial proteins were detected in CF, indicating that nuclear and mitochondria lysis did not occur, thus the CF extraction was successful. The purity can also be confirmed by ponceau S staining, as different pattern of protein band can be observed (Additional Data Fig. 3).

### RNA isolation

Total RNA was isolated by RNeasy/miRNeasy Mini Kit (Qiagen, Hilden, Germany) according to the manufacturer's protocol. For cytosolic RNA isolation, 700 μl of supernatant were mixed with 100% ethanol and transferred to an RNeasy spin column placed in a 2 ml collection tube. The column was centrifuged for 15 s at > 8000 g. The flow-through was discarded. The step was repeated to increase the RNA concentration. After washing with 500 μl of Buffer RW1, the RNA is treated with DNase I while bound to the RNeasy membrane. The DNase I is removed by a second wash with Buffer RW1. Washing with Buffer RPE and elution of RNA were then performed according to the standard protocols.

### Terminator 5′-phosphate-dependent exonuclease (TE) digestion

TE digestion was performed according to the manufacturer’s protocol. Briefly, 200–400 ng RNA were digested in 20 μL of reaction buffer containing 2 μL Terminator 10X reaction buffer A, 0.5 μL RiboGuard RNase Inhibitor and 1 μL Terminator Exonuclease. The mixture was incubated at 30 °C in a thermocycler for 60 min. The digested RNA was then purified and enriched by ethanol precipitation and RNeasy spin column as described above. TE digested treatment during immunofluorescence staining was performed after fixation. The slides with drop of digestion mixture were incubated in an oven at 30 °C for 60 min.

### Western blot

Isolation and culture of primary keratocytes were performed as previously described^1^. Samples were lysed in Radioimmunoprecipitation Assay (RIPA) lysis buffer, supplemented with protease inhibitor (Sigma, St. Louis, MO) and diluted in Laemmli buffer (Bio‐Rad, Hercules, CA) supplemented with β‐mercaptoethanol. After boiling the samples, equal total proteins, were loaded into each well of a pre‐made gel of 12% (Mini‐PROTEAN TGX, Bio‐Rad, Hercules, CA) and ran at 120 V for ≈60 min. Subsequently, proteins were transferred to a polyvinylidene fluoride transfer membrane (PVDF; Santa Cruz, Dallas, TX) for 60 min at 100 V. Membranes were blocked for 60 min in room temperature before primary antibody was added and incubated at 4 °C overnight. After washing, the membranes were exposed to the secondary antibody (conjugated with horseradish peroxidase, HRP) for 60 min. After additional washing the membrane was exposed to the enhanced chemiluminescence solution (GE healthcare, Little Chalfont, UK) for 5 min in room temperature. The membranes were developed using Odyssey Fc imaging system (LI‐COR, Lincoln, NE). All antibodies used are summarized in Table 2.

**Table 2:** Antibodies used for immunofluorescence staining and western blot.

Antibody	Company	Code	Species
β‐actin	Cell signaling	4967	Rabbit
NF-*κ*B p65	Cell signaling	8242	Rabbit
phospho-NF-*κ*B p65 (Ser536)	Cell signaling	3033	Rabbit
TFAM	Cell signaling	8076S	Rabbit
Histone	Cell signaling	7631	Rabbit
MDA5	Invitrogen	700360	Rabbit
RIG-I	Invitrogen	MA5-31715	Rabbit
RIG-I	Cell signaling	3743S	Rabbit
GAPDH	Cell signaling	5174	Rabbit
a-SMA	Abcam	5694	Rabbit
J2	Scicons		Mouse
Anti-rabbit IgG HRP‐linked	Cell signaling	7074	Rabbit
Anti-mouse IgG HRP‐linked	Cell signaling	7076	Mouse

### RT-qPCR

Isolation and culture of primary keratocytes were performed as previously described^1^. RNA was reverse transcribed into cDNA with High‐Capacity cDNA Reverse Transcription Kit (Life Technologies, Carlsbad, CA). To determine the gene expression, TaqMan Gene Expression Assays (Applied Biosystems, Carlsbad, CA) were used. cDNA transcribed from 40 ng of RNA was run in duplicates with the ViiA7 Real‐Time PCR system and analyzed with its software (Applied Biosystems, Carlsbad, CA). Gene expression was measured by using TaqMan Gene Expression Assay (Applied Biosystems, Carlsbad, USA). To quantify mtRNA, the same multiplex PCR setting as for mtDNA quantification was used (c.f. Multiplex qPCR for mtDNA and CD quantification). Results are presented as target gene expression/β‐actin normalized to the control group. All probes used for real‐time PCR (Applied Biosystems, Carlsbad, CA) are summarized in Table 3.

**Table 3:** Probes used for real‐time PCR from Applied Biosystems.

Gene name	Assay ID
Lumican	Hs00929860_m1
IL-8	Hs0017103_m1
NFKBIA	Hs00355671-g1
DDX58	Hs01061436_m1
IFIH1	Hs00223420_m1
Keratocan	Hs00559941_m1
Collagen I	Hs00164004_m1
Collagen V	Hs00609133_m1
β‐actin (reference gene)	4352667

### siRNA transfection

Silencing of genes of interest was performed using stealthRNA (Thermo Fisher Scientific) with Lipofectamine RNAiMAX (Thermo Fisher Scientific) in keratocytes according to the manufacturer’s instructions. The siRNA used were: HSS127414 and HSS127415 for IFIH1, HSS119010 and HSS177513 for DDX58, and 12935300 for the negative control. The stealthRNA oligonucleotides were used at a final concentration of 20 nM.

### Multiplex qPCR for mtDNA and CD quantification

Multiplex qPCR protocol was adapted from Phillips et al.^74^. Real-time PCR was performed on the ViiA7 Real‐Time PCR system and analyzed with its software (Applied Biosystems, Carlsbad, CA). PCR was performed using the following thermal profile: 95 °C for 20 s; 40 cycles of 95 °C for 1 s, 60 °C for 20 s. The reaction components are as follows: 10 μL Taqman Fast advanced Master Mix, each primer at 50 nM, and each dual hybridization probe at 250 nM. The final reaction volume is 20 μL. For CD quantification, the ND4 primers and probe set was substituted by the CD primers and probe set. Other conditions were the same. Primer sequence information is summarized in Table 4.

**Table 4:** Primers and probes used for multiplex qPCR real‐time PCR.

Primer name	Sequence (5′–3′)
7SF	CTAAATAGCCCACACGTTCCC
7SR	AGAGCTCCCGTGAGTGGTTA
ND4F	CTGTTCCCCAACCTTTTCCT
ND4R	CCATGATTGTGAGGGGTAGG
Fβ2M	GCTGGGTAGCTCTAAACAATGTATTCA
Rβ2M CD quantification primers^1^	CCATGTACTAACAAATGTCTAAAATGGT
HSSN8416	CCTTACACTATTCCTCATCACC
HSAS8542	TGTGGTCTTTGGAGTAGAAACC

Probe name	Sequence (5′–3′)
7SP	6FAM-CATCACGATGGATCACAGGT(NFQ)
ND4P	Texas Red-GACCCCCTAACAACCCCC(NFQ)
Pβ2MP (reference gene)	VIC-CAGCAGCCTATTCTGC(NFQ)
CD	Texas Red-TGGCAGCCTAGCATTAGCAGTT(NFQ)^72^

### Immunofluorescence staining

Cells were incubated with MitoTracker Red CMXRos (50 nM) for 30 min at 37 °C. Cells were then washed twice with cold PBS before fixed and permeabilized with precolded methanol for 2 h at − 20 °C. After the fixation, cells were washed twice with PBS and blocked with 5% PBS for 60 min at room temperature. After that cells were incubated with the J2 antibody (1:200) 60 min at room temperature. After washing, cells were incubated with secondary donkey anti-mouse IgG conjugated with Alexa Fluor 488 at (1:300) concentration for 60 min. Finally cells were washed three times with PBS and mounted with Prolong Diamond antifade mountant with DAPI (Invitrogen). A Zeiss Axioskop 2 plus microscope equipped with epifluorescence and an Olympus DP70 digital camera was used for analysis.

### ROS measurement

Cells cultured in six-well plate were labeled with DCFH-DA (5 µM) and MitoSOX (5 µM) in Serum-free DMEM/F‐12 media for 30 min. Subsequently, the cells were wash twice with PBS and harvested with trypsin and resuspended in 1 ml PBS. Flow Cytometry analysis was performed on FACSLSRII analysis machine and data was analyzed and illustrated by Flowing software (http://flowingsoftware.btk.fi/).

### ELISA

ELISA was performed as described previously^43^. 0.3 × 10^4^ corneal fibroblasts were seeded into 96-well plates in DMEM/F-12 supplemented with10% FBS for 24 h before treatment. Fibrosis was induced as described earlier. Desired wells were pretreated with either 10 μM mitoTEMPO or 20 nM TPCA. Supernatants were collected 2 days after treatments. Secretion of IL-8 was assessed with Human CXCL/IL-8 DuoSet (R&D Systems, #DY208), pro-collagen I was assessed with Human Pro-Collagen I alpha 1 DuoSet ELISA (R&D Systems, #DY6220), collagen III with Human Collagen, type III, alpha 1 (COL3A1) ELISA kit (Cusabio, Houston, TX, USA, # CSB-E13446h) and collagen V with Human Collagen, type V, alpha 1 (COL5A1) ELISA kit (Cusabio, # CSB-E13447h) according to the manufacturer protocol.

### Hydroxyproline assay

Cells were treated the same as described in ELISA assay. Supernatants were collected 2 days after treatments. A hydroxyproline assay was used to determine the free hydroxyproline content in each group. This offered further insights on incorporation of free hydroxyproline in collagen synthesis. This assay was performed on hydrolyzed samples using a hydroxyproline assay kit (Abcam, Cambridge, UK). Briefly, supernatants were mixed with concentrated NaOH (10 N) in a tightened screw-capped polypropylene vial and then heated at 120 °C for 60 min. The alkaline lysate was cooled on ice, neutralized, and centrifuged to obtain supernatants. The hydrolysates were dried, oxidized, and finally reacted with 4-(dimethylamino) benzaldehyde. The resultant colored product was detected at 560 nm and found to be proportional to the hydroxyproline present in the sample.

### RNA-seq

Total RNA was collected from cells from six unique donors (n = 6): Three donors with CD+ cells and three donors with CD− cells. From each biological donor, RNA was collected in three technical replicates. The total RNA from CD+/− cells were pooled together in each group, prior to sequencing. Three repeats were sequenced in each group (n = 3). Sequencing was performed at Novogene Co., Ltd. using the Illumina HiSeq 2500 instrument. RNA-Seq data was aligned to the reference genome (human assembly GRCh37/hg19) using Tophat2 (http://ccb.jhu.edu/software/tophat). HTSeq (http://www-huber.embl.de/HTSeq) was then applied on the aligned data set to determine differentially expressed genes with a “significant” status. The resulting P-values were adjusted using the Benjamini and Hochberg’s approach for controlling the false discovery rate. Genes with an adjusted P-value of < 0.05 were assigned as differentially expressed. The Gene Ontology and KEGG analyses of the differentially expressed genes were performed using DAVID (https://david.ncifcrf.gov/). The KEGG graph was rendered by Pathview^75^. Volcano plot was generated by BioJupies (http://biojupies.cloud.). Gene fold changes were transformed using log2 and displayed on the x-axis: P-values were corrected using the Benjamini–Hochberg method, transformed using –log10, and displayed on the y axis. Quality report and more detailed analysis can be downloaded from the following link: https://drive.google.com/drive/folders/1Kup4CKnaAotz2fuoF19rzSC7R7Sz1ha7?usp=sharing. The RNA-seq raw data is provided upon reguest.

### Statistics

Statistical analysis was performed using Student's t‐test when comparing two groups. One‐way ANOVA with Tukey's multiple comparisons was performed when comparing between more than two groups. Differences were considered statistically significant at a *P*‐value of < 0.05. All experiments were repeated successfully at least three times (i.e., at least three separate experiments were performed with cells isolated from different donors). All experimental samples were prepared in triplicates.

### Ethical approval and consent to participate

Not applicable.

### Consent for publication

Not applicable.

## Supplementary Information

Supplementary Information.

## Data Availability

Data and materials related to this study are available from the corresponding author on reasonable request.
