# Supplementary material for: Activation of NF-κB signaling via cytosolic mitochondrial RNA sensing in kerotocytes with mitochondrial DNA common deletion
Source: Sci Rep. 2021 Apr 1;11:7360. doi: 10.1038/s41598-021-86522-6 (PMC8016944; doi:10.1038/s41598-021-86522-6)
Supplement: Supplementary file 1 — Supplementary Information. [file 41598_2021_86522_MOESM1_ESM.docx]

**Activation of NF-κB signaling via cytosolic mitochondrial RNA sensing in kerotocytes with mitochondrial DNA common deletion**

Xin Zhou ^a*^, Ludvig J Backman ^a, c^, Patrik Danielson ^a,b*^

^a^Department of Integrative Medical Biology, Umeå University, 90187 Umeå, Sweden

^b^Department of Clinical Sciences, Ophthalmology, Umeå University, Umeå, Sweden

^c^Department of Community Medicine and Rehabilitation, Physiotherapy, Umeå University, 90187 Umeå, Sweden

* Corresponding authors: Xin, Zhou, Patrik Danielson.

**Email:**  [xin.zhou@umu.se](mailto:xin.zhou@umu.se), [patrik.danielson@umu.se](mailto:patrik.danielson@umu.se)

**Additional Data**


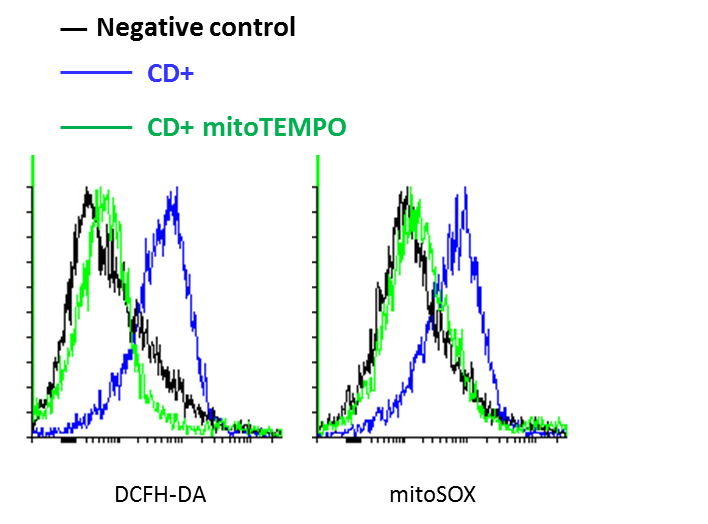


Additional Data Figure 1. Flow cytometric detection of DCFH-DA and mitoSOX signal in CD+ cells treated with mitoTEMPO (*n*=3).


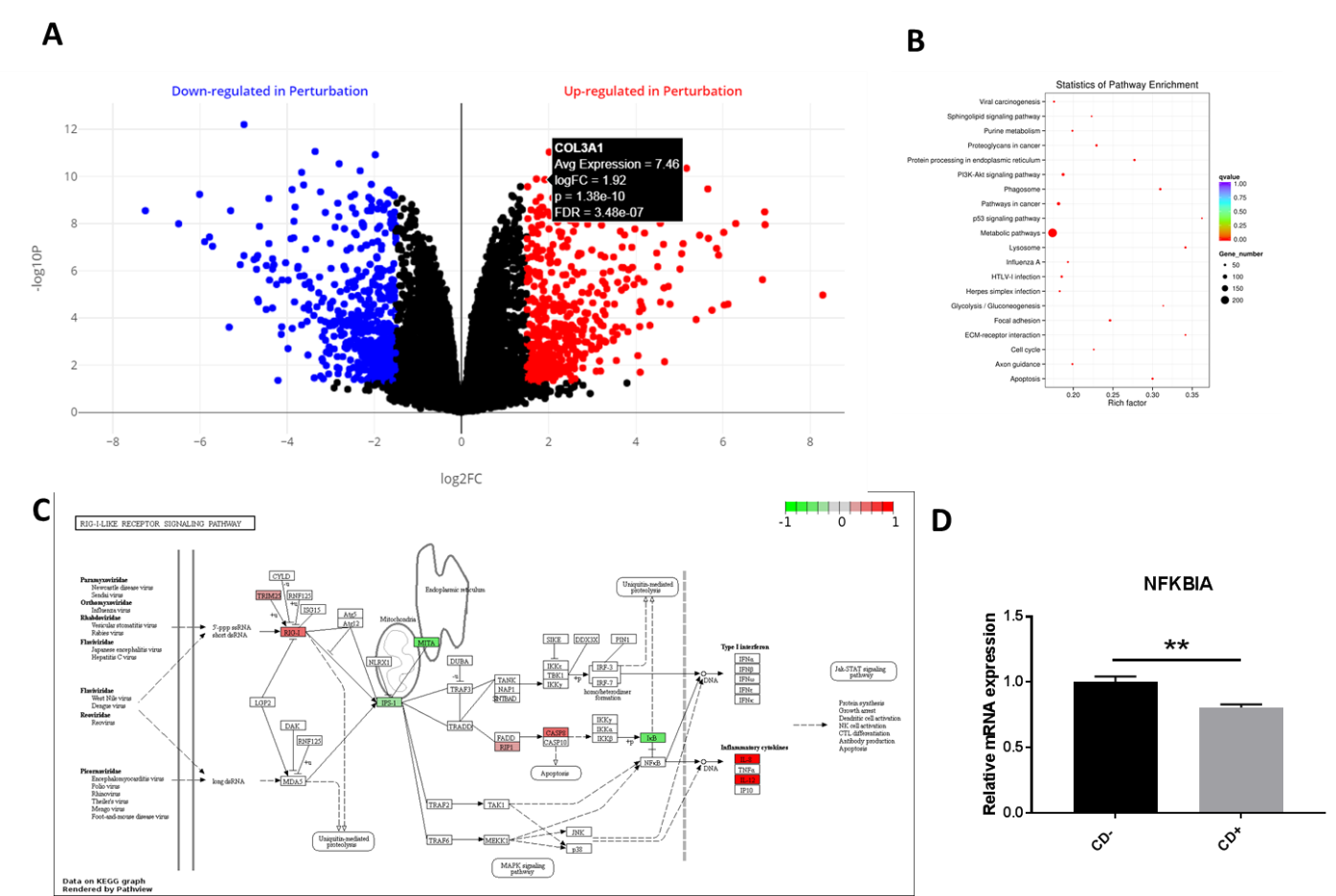


Additional Data Figure 2. (A) Volcano plot for differentially expressed genes significantly up and down regulated genes are highlighted in red and green, respectively. (B) KEGG Enrichment Scattered Plot in CD+ cells compared with CD- cells. (C) Diagram showing significantly upregulated/downregulated genes in RIG-I-like receptor signaling pathway. (D) NFKBIA mRNA expression in CD+/- keratocytes (*n*=3). Source data of enrichment and pathway analysis are generated by KEGG ^1-3^.


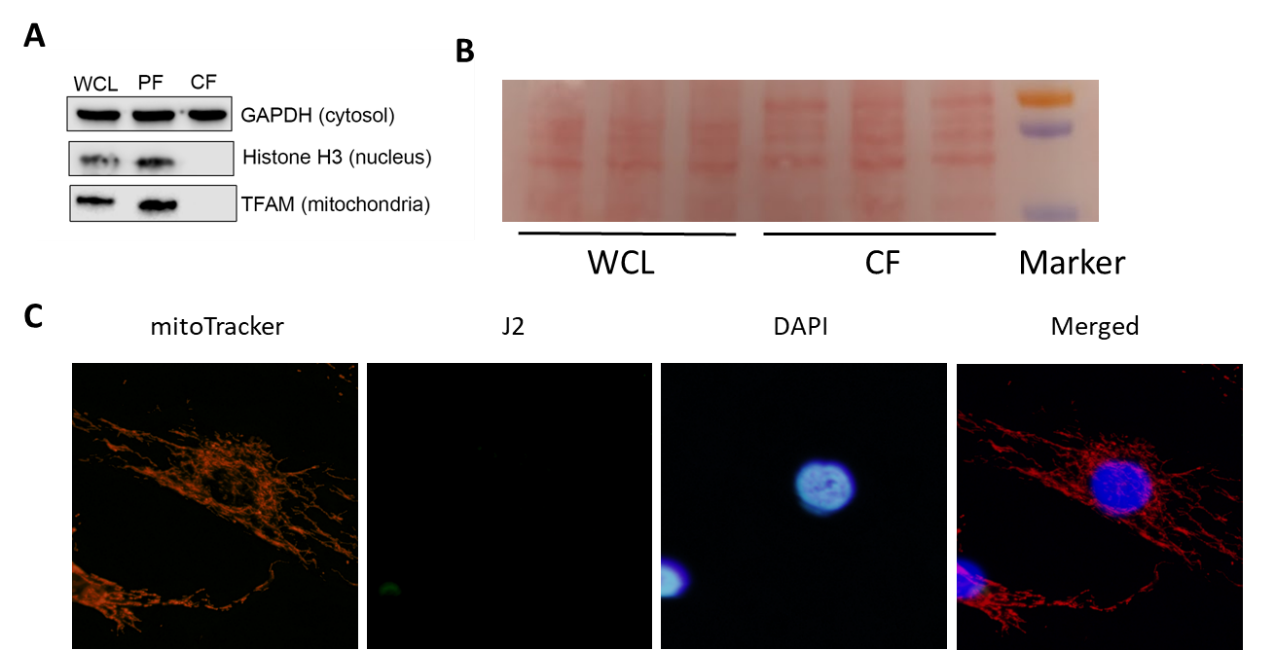


Additional Data Figure 3. (A) Keratocytes were subjected to digitonin fractionation as described in the Methods and whole-cell extracts (WCL), pellets (PF) or cytosolic extracts (CF) were blotted using the indicated antibodies (*n*=3). (B) Ponceau S staining of WCL and CF from three different samples (*n*=2). (C) Representative fluorescence image of dsRNA in keratocytes treated with TE digestion before J2 staining.


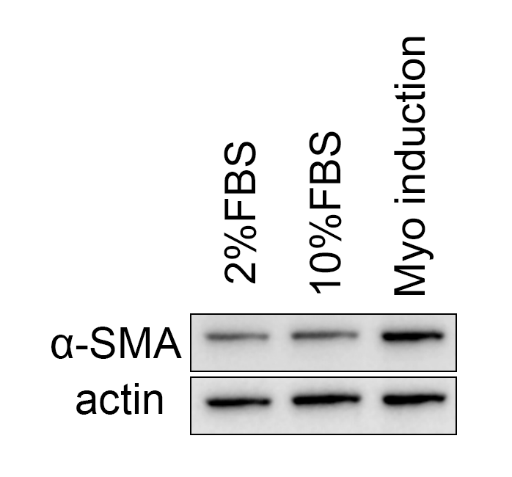


Additional Data Figure 4. α-SMA protein expression was assessed by western blot at 2 d in F-12 medium containing 2% FBS, 10% FBS or stimulated for myofibroblast induction as described in M&M.


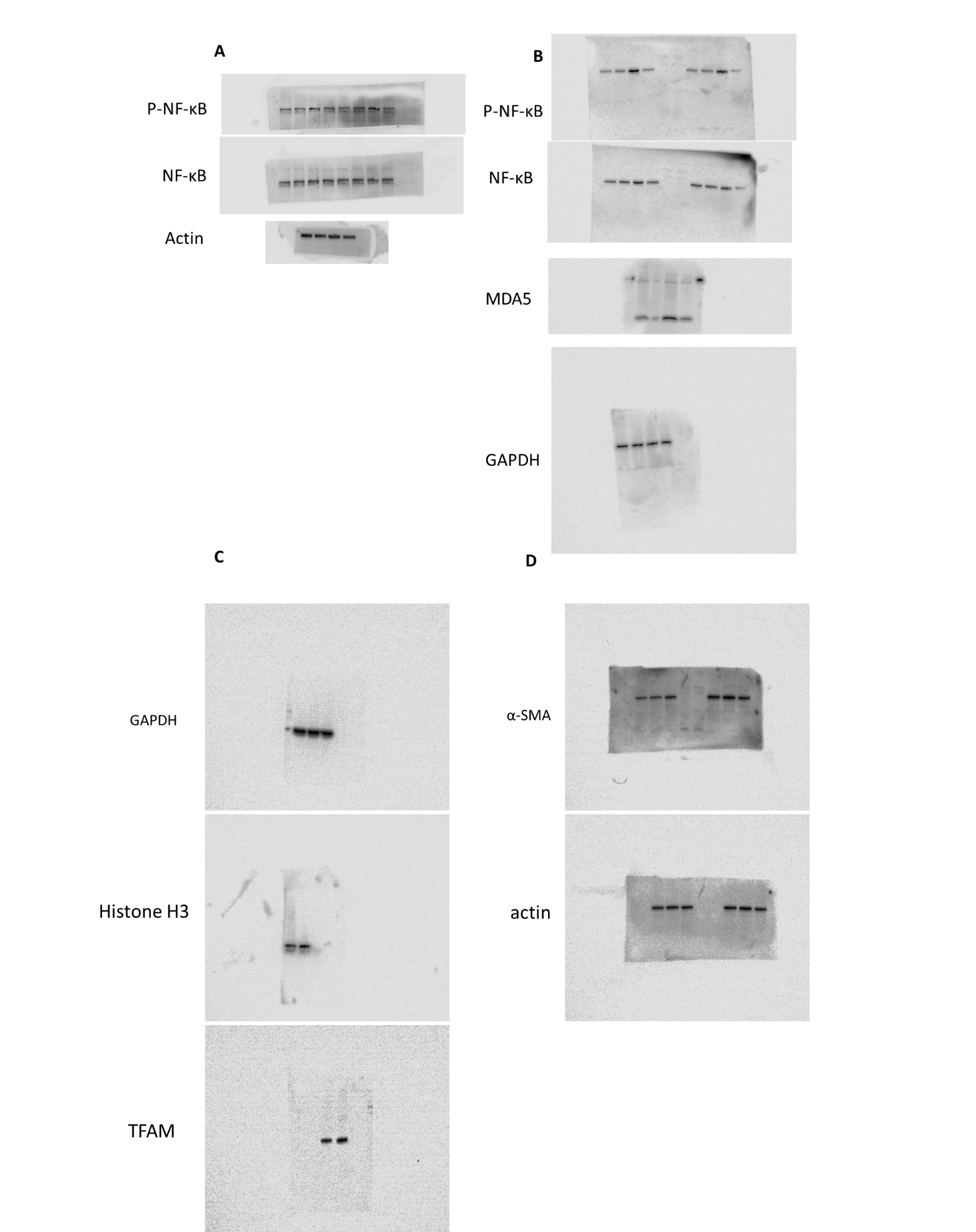


Additional Data Figure 5. Full-length blots of Figure 2A(A), Figure 2D (B), Additional Data Figure 3A(C) and Additional Data Figure 4(D).

1. Kanehisa, M. & Goto, S. KEGG: kyoto encyclopedia of genes and genomes. *Nucleic Acids Res* **28**, 27-30 (2000).

2. Kanehisa, M. Toward understanding the origin and evolution of cellular organisms. *Protein Sci* **28**, 1947-1951 (2019).

3. Kanehisa, M., Furumichi, M., Sato, Y., Ishiguro-Watanabe, M. & Tanabe, M. KEGG: integrating viruses and cellular organisms. *Nucleic Acids Res* (2020).
